# *Syzygium oleosum* (F.Muell.) B.Hyland (Myrtaceae), an Unexplored Australian Species: Anatomical and Micromorphological Study of Leafy Twigs, and Characterization and Biological Activity of Their Essential Oil

**DOI:** 10.3390/plants14162605

**Published:** 2025-08-21

**Authors:** Paola Malaspina, Flavio Polito, Susanna Alloisio, Raffaella Barbieri, Greg Trevena, Eleonora Agostino, Domenico Trombetta, Vincenzo De Feo, Laura Cornara, Antonella Smeriglio

**Affiliations:** 1 Department of Earth, Environment and Life Sciences (DISTAV), University of Genova, Corso Europa 26, 16132 Genova, Italy; paola.malaspina@unige.it; 2 Department of Pharmacy (DIFARMA), University of Salerno, Via Giovanni Paolo II 132, 84084 Fisciano, Italy; fpolito@unisa.it (F.P.); defeo@unisa.it (V.D.F.); 3 ETT S.p.A., Via Albareto 21, 16153 Genova, Italy; susanna.alloisio@dedagroup.it; 4 Biophysics Institute of the Italian National Research Council, CNR-IBF, Via De Marini 6, 16149 Genova, Italy; raffaella.barbieri@ibf.cnr.it; 5 Essentially Australia, Unit 1/7 Sunrise Boulevard, Byron Bay, NSW 2481, Australia; info@essentiallyaustralia.com.au; 6 Department of Chemical, Biological, Pharmaceutical and Environmental Sciences (ChiBioFarAm), University of Messina, Viale Ferdinando Stagno d’Alcontres 31, 98166 Messina, Italy; eleonora.agostino@unime.it (E.A.); domenico.trombetta@unime.it (D.T.); antonella.smeriglio@unime.it (A.S.)

**Keywords:** Mango Myrtle, micromorphology, essential oil, phytochemistry, antioxidant activity, anti-inflammatory activity, acetylcholinesterase, butyrylcholinesterase, GABA transaminase, neuromodulation

## Abstract

*Syzygium oleosum* (F.Muell.) B.Hyland is an Australian native species whose essential oil (EO), known commercially as “Mango Myrtle,” is gaining popularity in aromatherapy, yet remains poorly studied. This work provides the first comprehensive pharmacognostic investigation of *S. oleosum*. Anatomical and micromorphological analyses revealed numerous secretory cavities and calcium oxalate druses in both leaves and twigs. GC-MS analysis identified 16 components in the EO, predominantly hydrocarbon monoterpenes, with terpinolene (30.79%), β-pinene (26.79%), α-pinene (10.69%), and γ-terpinene (9.86%) as major constituents. In vitro assays showed moderate antioxidants (IC_50_ ≤ 4.95 mg/mL) and anti-inflammatory effects (IC_50_ ≤ 5.93 mg/mL), with specific monoterpenes contributing differentially to each activity. The EO displayed weak inhibitory activity against acetylcholinesterase (IC_50_ 19.4 mg/mL) and butyrylcholinesterase (IC_50_ 15.9 mg/mL), and no effect on GABA transaminase. Microelectrode array recordings on primary cortical neurons demonstrated a concentration-dependent inhibition of network activity (0.059–1.19 mg/mL) without affecting cell viability, indicating a neuromodulatory property. These results provide new insights into the pharmacological potential of *S. oleosum* EO and support its further evaluation as a neuroactive and anti-inflammatory agent.

## 1. Introduction

The genus *Syzygium*, a member of the Myrtaceae family, comprises over a thousand species predominantly distributed across tropical and subtropical regions of the world, particularly in Asia, Oceania, Africa, and certain areas of the Americas [1,2,3]. This genus encompasses both small shrubs and large trees [4], which are appreciated for their vibrant, fragrant flowers—enhancing their ornamental appeal—and for their sweet, edible fruits [3,5,6].

A comprehensive phytochemical review by Kadir et al. [7] revealed that essential oils (EOs) from various *Syzygium* species typically contain both sesquiterpenes—such as α- and β-caryophyllene, caryophyllene oxide, α-cadinol, germacrene D, viridiflorol, and nerolidol—and monoterpenes, including α-pinene, β-pinene, o-cymene, β-ocimene, and limonene. The relative abundance of these classes varies across species, with sesquiterpenes often representing the dominant fraction, while monoterpene hydrocarbons can also occur in significant amounts. Both groups of compounds contribute to the sensory characteristics of the EOs and may underlie several of the biological effects observed for this genus. Several *Syzygium* species are also recognized for their substantial therapeutic potential, exhibiting a broad range of biological activities including anti-inflammatory, anti-allergic, antimicrobial, anti-ulcer, anti-diabetic, hepatoprotective, and antioxidant properties [3,8,9].

Specifically, compounds such as (E)-β-caryophyllene, β-pinene, and (E)-β-ocimene have been associated with marked anti-cholinesterase, antidiabetic, and antioxidant activities in various *Syzygium* species [10].

Among the lesser-known members of this genus is *Syzygium oleosum* (F.Muell.) B. Hyland, which has been described recently in Australian Journal of Botany Supplementary Series (1983). Its aboriginal name is Wanduin (Ganai), but it is commonly referred to as “Blue Lilly Pilly” or “Blue Cherry.” This evergreen shrub or tree is native to Australia, where it grows naturally along the eastern coastlines of New South Wales (NSW) and Queensland. Its distinctive blue to purple berries, noted for their sweet and mildly spicy flavor, are consumed fresh or processed into jams [11,12,13].

Despite its underutilized status, *S. oleosum* shares ecological and morphological similarities with other Australian native species such as *Syzygium australe* (J.C.Wendl. ex Link) B.Hyland, which has shown potent antidiabetic and antioxidant activities [14].

In 2016, a commercial plantation was established near Byron Bay (NSW) to cultivate *S. oleosum* for EO production from its leafy branches [15]. This EO is characterized by a unique and pleasant mango-like or green mango aroma and is marketed under the name “Mango Myrtle.” It is increasingly used in potpourri, air fresheners, and cosmetic products [16]. Despite its growing commercial relevance, the potential health-promoting properties of this EO remain largely unexplored.

Given the well-established role of EOs in aromatherapy, complementary, and traditional medicine—particularly for their effects on the central nervous system (CNS)—it is important to investigate the biological activities of *S. oleosum* EO.

Indeed, a growing body of research has demonstrated that EOs can modulate CNS function through a variety of mechanisms, including anxiolytic, neuroprotective, antidepressant, anticonvulsant, analgesic, and sedative effects [17].

Essential oils, predominantly composed of lipophilic compounds, can cross the blood–brain barrier and exerting various neuroactive effects, including anti-inflammatory, antioxidant, anxiolytic, antidepressant, and anticonvulsant actions [18,19,20]. Previous studies have demonstrated that extracts and EOs from other *Syzygium* species possess CNS activity and antioxidant potential [9,10,21,22,23,24,25,26].

This pharmacological potential has been attributed to the presence of neuroactive terpenes capable of modulating neurotransmitter systems, reducing oxidative stress, and enhancing synaptic plasticity [18,19,20]. However, the biological properties of the EO derived from *S. oleosum* twigs and leaves have been only minimally investigated.

Considering its economic importance and endemic nature, the present study aimed to conduct a comprehensive pharmacognostic investigation of *S. oleosum*. Specifically, we analyzed the micromorphological and anatomical characteristics of twigs and leaves, and determined the phytochemical composition and potential antioxidant, anti-inflammatory, and neuromodulatory effects of their EO. These findings contribute to the scientific knowledge of this underexplored species and may support the future therapeutic applications of its EO.

## 2. Results

### 2.1. Anatomical and Micromorphological Studies

**Figure 1:**
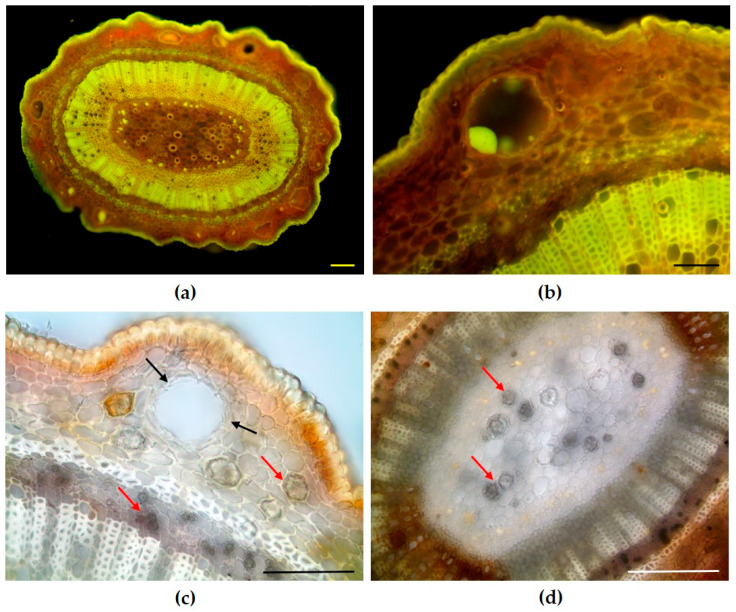
Light microscopy images of twigs of *Syzygium oleosum* (F.Muell.) B.Hyland. (**a**,**b**) Transverse sections stained with Fluorol Yellow showing numerous secretory cavities in the cortex (**a**), and a magnified view of a secretory cavity in which the secretion exhibited a positive reaction to the dye, revealing its lipophilic nature (**b**). (**c**,**d**) Cleared hand-cut transverse sections highlighting a secretory cavity lined by a single epithelial layer of secretory cells ((**c**), black arrows), and the distribution of calcium oxalate druses ((**c**,**d**), red arrows). Scale bars = 100 µm.

In a cross-section, the twig exhibited an ovoid outline and revealed numerous secretory cavities arranged in a circular pattern within a multilayered cortex (Figure 1a). The presence of lipophilic compounds within these cavities was evidenced by the bright yellow-green fluorescence following Fluorol Yellow staining (Figure 1b). The cavities were in fact lined by an epithelium composed of secretory cells (Figure 1c, black arrows), responsible for producing EO. Numerous druse-type crystals were observed in the cortex near the secretory cavities, as well as scattered throughout the phloem (Figure 1c, red arrows), and present in the parenchymatous pith (Figure 1d, red arrows).

**Figure 2:**
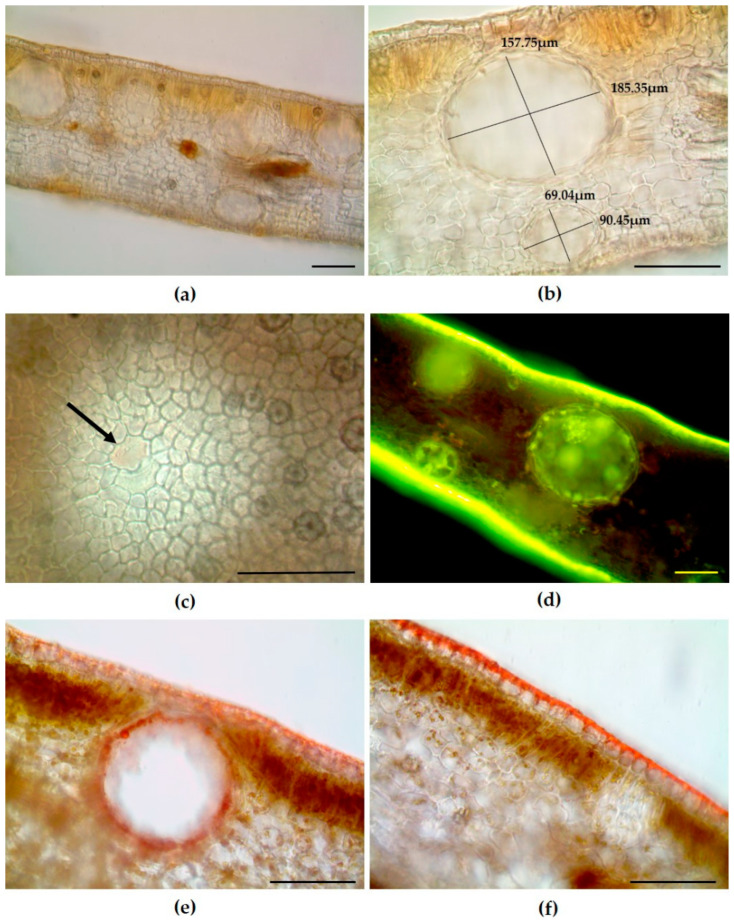
Light microscopy images of leaves of *Syzygium oleosum* (F.Muell.) B.Hyland. (**a**) Cleared hand-cut transverse sections showing the presence of secretory cavities distributed throughout the mesophyll. (**b**) Magnified view of two secretory cavities highlighting size variation, with larger cavities located near the upper epidermis compared to those near the lower epidermis. (**c**) Cleared epidermal surface revealing two overlying epidermal cells situated above a secretory cavity (arrow). (**d**) Transverse section stained with Fluorol Yellow indicating the lipophilic nature of both the cuticle and the secretion within the secretory cavities. (**e**,**f**) Transverse sections stained with Sudan III showing positive staining of the secretory cells lining the cavity (**e**) and of the thick cuticle covering the uniseriate epidermis (**f**). Scale bars = 100 µm.

The leaf cross-section of *S. oleosum* displayed a uniseriate epidermis and a dorsiventral organization, with a bilayered mesophyll in which the spongy tissue occupied most of the leaf thickness (Figure 2a). Secretory cavities, each lined with a secretory epithelium (Figure 2b), were distributed throughout the mesophyll and were generally more numerous and more prominently protruding toward the upper epidermis than the lower one (Figure 2a). Variations in their size were also noted (Figure 2b and Table 1).

Observations of cleared leaf epidermal surfaces revealed the presence of two overlying epidermal cells situated above each secretory cavity (Figure 2c, arrow). Each cavity was surrounded by a single epithelial layer, and both the secretory cells and their contents showed positive staining with Fluorol Yellow (Figure 2d) and Sudan III (Figure 2e), indicating the presence of lipophilic substances. Furthermore, a thick cuticular layer was detected on both epidermal surfaces, exhibiting bright yellow fluorescence with Fluorol Yellow and an orange-red staining with Sudan III (Figure 2d and Figure 2f, respectively).

**Figure 3:**
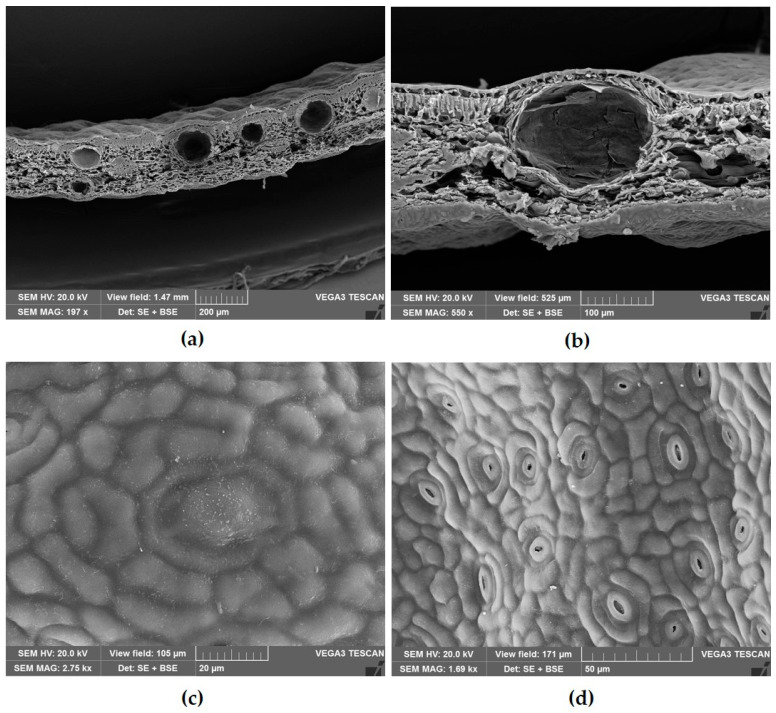
Scanning electron microscopy images of transverse sections and epidermal surfaces of leaves of *Syzygium oleosum* (F.Muell.) B.Hyland. (**a**) Transverse section showing secretory cavities distributed within the mesophyll. (**b**) Higher magnification of a secretory cavity. (**c**) Epidermal surface showing enlarged overlying cells covering the secretory cavities. (**d**) Leaf surface displaying mainly paracytic stomata.

Scanning electron microscopy (SEM) analysis confirmed the presence of numerous secretory cavities within the mesophyll, predominantly located near the upper epidermis (Figure 3a,b). The overlying epidermal cells displayed distinct differences in shape and size compared to surrounding epidermal cells (Figure 3c). Additionally, *S. oleosum* was found to possess hypostomatic leaves with mainly paracytic stomata, and occasionally the cyclostaurocytic ones as well (Figure 3d).

**Figure 4:**
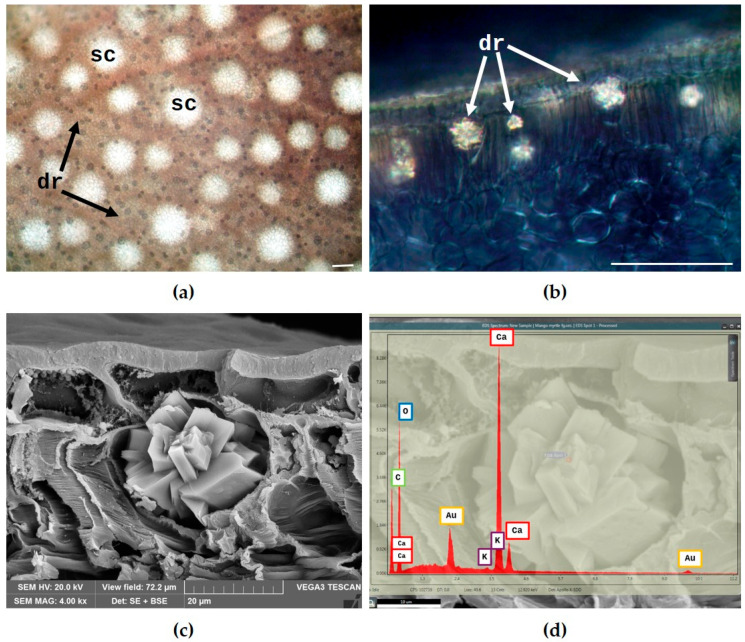
(**a**) Cleared leaf surface of *Syzygium oleosum* (F.Muell.) B.Hyland, showing under the epidermis numerous calcium oxalate druses (dr) scattered around the secretory cavities (sc). (**b**,**c**) Hand-cut transverse sections of leaf and twig observed under polarized light using LM. (**b**) Druses primarily located in the palisade parenchyma of the leaf. (**c**) Distribution of crystal druses within the twig (arrows). (**d**) SEM–EDS structural characterization of a calcium oxalate druse within the leaf parenchyma. The “Au” peak corresponds to the gold coating applied to the sample. The insert shows an analyzed crystal druse.

Finally, numerous druse crystals were detected in both the leaf and twig tissues using light microscopy (LM) (Figure 4a–c). Druses (dr) were clearly visible on cleared leaf surfaces (Figure 4a, arrows) scattered around the secretory cavities (sc). Their presence was further highlighted in cleared transverse sections observed under polarized light, where they appeared primarily located within the palisade parenchyma (Figure 4b, arrows). Observation under polarized light also confirmed the presence and distribution of crystal druses in the twig, consistent with previous findings (Figure 4c).

The chemical composition of these crystals was determined using SEM coupled with energy-dispersive spectroscopy (SEM-EDS). The analysis identified them as calcium oxalate druses, as indicated by the prominent calcium peak in the EDS spectrum (Figure 4d).

### 2.2. Chemical Composition of the Essential Oil

Hydrodistillation of the *S. oleosum* leaves yielded a light lime-green to yellow EO with an average yield of approximately 0.75%.

The GC chromatogram of the EO, showing the separation profile of the volatile constituents, is reported in Figure S1. Sixteen components were identified in the EO, accounting for 100.00% of the total composition (Table 2). Most of the identified constituents belonged to the class of monoterpene hydrocarbons (94.25%), followed by oxygenated monoterpenes (5.18%) and sesquiterpene hydrocarbons (0.57%). The most abundant components were terpinolene (30.79%), β-pinene (26.79%) and α-pinene (10.69%). Other notable constituents included γ-terpinene (9.86%) and α-thujene (4.07%), all of which are monoterpene hydrocarbons, confirming the predominance of this class in the EO.

Among the oxygenated monoterpenes, the most representative compounds were terpinen-4-ol (2.26%) and α-terpineol (2.11%). The only sesquiterpene hydrocarbon detected was β-caryophyllene, present at a low concentration (0.57%).

### 2.3. Antioxidant and Anti-Inflammatory Properties

The antioxidant and anti-inflammatory properties of *S. oleosum* EO were investigated through a panel of in vitro assays, with the aim of elucidating the contribution of its most abundant monoterpene constituents—namely α-pinene, β-pinene, γ-terpinene, and terpinolene—to the overall biological activity (Table 3). All results are expressed as IC_50_ values, where lower concentrations correspond to higher efficacy in inhibiting the targeted oxidant or inflammatory processes.

The antioxidant activity was investigated by the following assays: Trolox Equivalent Antioxidant Capacity assay (TEAC), Ferric Reducing Antioxidant Power assay (FRAP), Oxygen Radical Absorbance Capacity assay (ORAC), β-Carotene Bleaching test (BCB), and Iron-Chelating Activity assay (ICA). The EO exhibited moderate radical-scavenging capacity in the TEAC assay (IC_50_ = 4.95 mg/mL). On the contrary, none of the pure compounds reached 50% inhibition at the tested concentrations, indicating negligible activity in this model. The same behavior was detected also for FRAP assay. The ORAC assay, which evaluates peroxyl radical scavenging through a hydrogen atom transfer mechanism, revealed strong antioxidant activity for EO (IC_50_ = 0.02 mg/mL), with comparable potency observed for terpinolene (0.01 mg/mL). The remaining compounds did not reach 50% inhibition within the tested range, indicating lower scavenging capacity under the same conditions. BCB assay, which mimics lipid peroxidation, highlighted a strong inhibitory effect by γ-terpinene (IC_50_ = 0.02 mg/mL) and, to a lesser extent, by terpinolene (0.21 mg/mL), both of which outperformed the EO (0.24 mg/mL). α-Pinene and β-pinene were inactive in this model. These findings underscore the relevance of unsaturated structures with conjugated double bonds, such as those in γ-terpinene, for lipid-targeted antioxidant activity. Notably, in the ICA assay, β-pinene exhibited exceptional potency (IC_50_ = 0.03 mg/mL), followed by EO (0.12 mg/mL) and α-pinene (0.5 mg/mL). γ-Terpinene and terpinolene showed no appreciable activity. These results suggest that compact, hydrophobic, and strained bicyclic structures, such as that of α- and β-pinene, may facilitate interaction with metal ions and enhance chelating efficiency. In all antioxidant assays, however, the reference standards (Trolox for TEAC, FRAP, and ORAC; BHT for BCB; EDTA for ICA) consistently exhibited much lower IC_50_ values in the µg/mL range, confirming their markedly higher potency compared to both EO and its individual constituents.

Anti-inflammatory activity was also evaluated using two complementary models: the inhibition of protein denaturation (ADA) and the inhibition of protease activity (PIA) (Table 3). In the ADA assay, the strongest inhibitory effect was observed for γ-terpinene (IC_50_ = 0.11 mg/mL), followed by terpinolene (0.32 mg/mL) and β-pinene (0.36 mg/mL), all of which were more active than EO (3.53 mg/mL). In contrast, α-pinene did not reach 50% inhibition at the tested concentrations. This suggests that while each constituent possesses distinct potency, their combined action within the EO matrix may contribute to a moderate overall effect. Nevertheless, the reference standard diclofenac sodium displayed an IC_50_ of 17.05 µg/mL, highlighting a markedly stronger anti-denaturation effect than both EO and its components.

The PIA assay provided a different pattern of activity. α-Pinene emerged as the most effective inhibitor (IC_50_ = 0.13 mg/mL), followed by EO, which showed only modest activity (5.93 mg/mL). β-Pinene, γ-terpinene, and terpinolene did not reach the IC_50_ threshold. The high potency of α-pinene in this context may be explained by its physicochemical properties, which likely favor binding to the enzyme’s active site. Again, diclofenac sodium, used as reference standard, proved to be far more potent (IC_50_ = 28.50 µg/mL) than either EO or the pure compounds.

Overall, these data reveal that *S. oleosum* EO exerts a multifaceted antioxidant and anti-inflammatory effect, derived from the combined action of several major constituents. Specific activities can be attributed to distinct compounds—such as γ-terpinene in lipid peroxidation inhibition, β-pinene in metal chelation, and α-pinene in protease inhibition—while other effects appear to result from additive or synergistic interactions within the phytocomplex. These findings highlight a clear relationship between chemical structure and bioactivity, where the presence and position of double bonds, ring strain, and molecular conformation significantly influence the pharmacological potential of each compound.

### 2.4. Effect on Neuronal Function

Following the in vitro assessment of antioxidant and anti-inflammatory activities, the study further investigated the potential neuromodulatory properties of the EO and its major pure constituents. Specifically, their inhibitory effects were evaluated against key enzymes involved in neurotransmission, namely acetylcholinesterase (AChE), butyrylcholinesterase (BChE), and γ-aminobutyric acid transaminase (GABA-T).

Neither the tested EO nor its most abundant constituents exhibited any inhibitory activity against GABA-T under the experimental conditions. On the contrary, the inhibitory activity of EO against AChE and BChE, as well as the contribution of its main pure components to the potential anti-enzyme activity, are shown in Table 4.

The results are reported as IC_50_ values, with lower concentrations indicating greater inhibitory potency against the target enzymes. In the AChE inhibition assay, the EO exhibited modest activity (IC_50_ = 19.4 mg/mL), markedly lower than that of the reference compound galantamine (IC_50_ = 2.3 µg/mL). Among the individual constituents, α-pinene displayed the highest inhibitory activity (IC_50_ = 0.05 mg/mL), suggesting a major contribution to the observed enzymatic inhibition. Terpinolene also demonstrated appreciable activity (IC_50_ = 1.0 mg/mL), albeit to a lesser extent than α-pinene. In contrast, β-pinene and γ-terpinene did not achieve 50% inhibition within the concentration range tested, indicating a minimal or negligible role in the overall effect.

With respect to BChE, the EO showed slightly greater inhibitory activity (IC_50_ = 15.9 mg/mL) compared to its effect on AChE yet remained significantly less active than galantamine (IC_50_ = 10.4 µg/mL). Among the tested monoterpenes, terpinolene exhibited the strongest inhibitory effect against BChE (IC_50_ = 1.4 mg/mL), while α-pinene, β-pinene, and γ-terpinene failed to reach the IC_50_ threshold.

Overall, data suggest that the cholinesterase inhibitory activity of the EO was primarily attributable to synergistic or additive interactions among specific monoterpenes. Nevertheless, the inhibitory potency of some individual constituents exceeded that of the whole EO. In particular, α-pinene and terpinolene were more effective against AChE, while only terpinolene showed notable activity against BChE, highlighting the possibility of a selective inhibitory effect by individual compounds. This selectivity is likely related to structural differences among the molecules and their distinct modes of interaction with the active or allosteric sites of the target enzymes.

Considering these findings and given that neither the tested EO nor its most abundant constituents exhibited any inhibitory activity against GABA-T under the experimental conditions, subsequent cell-based studies were conducted exclusively with the EO, as it better represents the potential additive/synergistic interactions responsible for the observed biological effects.

**Figure 5:**
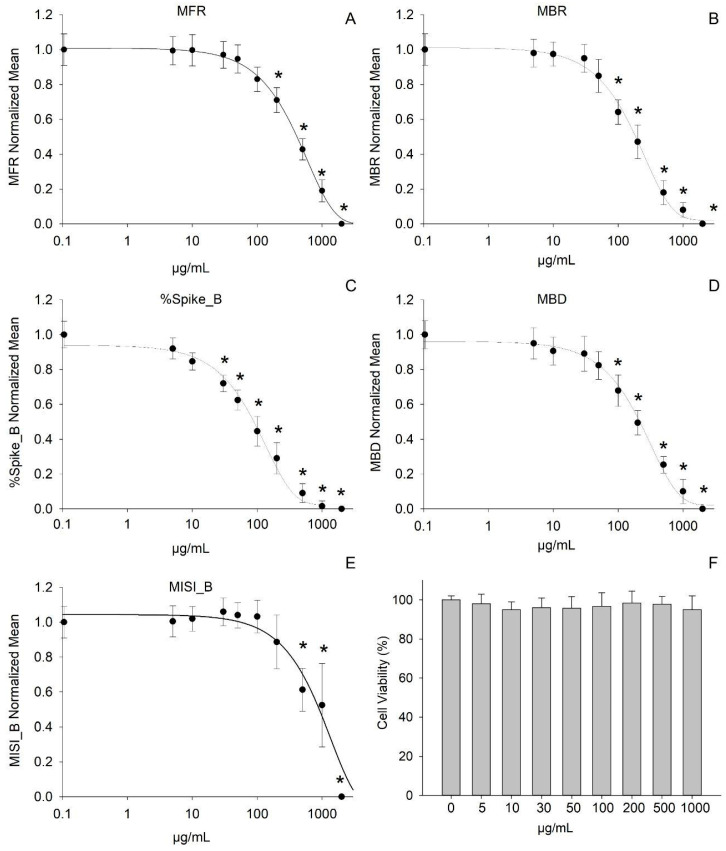
Effect of increasing concentrations of EO (5–2000 μg/mL) of *Syzygium oleosum* (F.Muell.) B.Hyland on neuronal network activity and cell viability (5–1000 μg/mL). A concentration-dependent modulation was observed across all evaluated parameters of neuronal network activity: (**A**) mean firing rate (MFR), (**B**) mean burst rate (MBR), (**C**) percentage of spikes within bursts (%Spike_B), (**D**) mean burst duration (MBD; s), and (**E**) mean inter-spike interval within bursts (MISI_B; s). Among these, %Spike_B was the most sensitive to EO exposure, whereas MISI_B was the least affected (*n* = 8);. (**F**) Bar graph showing that EO exposure did not significantly alter neuronal viability, as determined by the MTT assay (*n* = 12). Each data point is the mean and standard error of the mean (S.E.M.) (* *p* < 0.05 with respect to normalized baseline values).

The cytotoxicity of the EO was assessed by evaluating the viability of primary cortical neurons exposed to increasing concentrations of EO (5–2000 µg/mL). The MTT assay showed no significant reduction in cell viability across the tested concentration range, indicating an absence of overt cytotoxic effects (Figure 5F).

The effects of the EO on neuronal network function were subsequently examined using microelectrode array (MEA) recordings. The EO was applied cumulatively at increasing concentrations (5, 10, 30, 50, 100, 200, 500, 1000, and 2000 µg/mL), with each treatment maintained for 20 min. A concentration-dependent reduction in spontaneous network activity was observed, reflected by alterations in several electrophysiological parameters: mean firing rate (MFR), mean burst rate (MBR), percentage of spikes within bursts (%Spike_B), mean burst duration (MBD), and mean inter-spike interval within bursts (MISI_B).

The concentration–response analysis yielded the following IC_50_ values: MFR = 377.32 µg/mL; MBR = 171.03 µg/mL; %Spike_B = 59.30 µg/mL; MBD = 224.69 µg/mL; MISI_B = 1186.64 µg/mL.

The results indicate that the EO induces a progressive and selective inhibition of neuronal network activity. Among the parameters analyzed, %Spike_B exhibited the highest sensitivity (IC_50_ = 59.30 µg/mL), suggesting interference with the fine structure of bursting activity at relatively low concentrations. MBR and MBD were also affected in a concentration-dependent manner, indicating a gradual disruption of rhythmic burst dynamics.

Conversely, MFR was less sensitive (IC_50_ = 377.32 µg/mL), suggesting that overall spiking activity is more resistant to EO exposure. MISI_B was the least-affected parameter (IC_50_ = 1186.64 µg/mL), indicating that the temporal spacing of spikes within bursts remains relatively stable even at higher concentrations where burst structure is compromised.

## 3. Discussion

Several species of the genus *Syzygium*, belonging to the Myrtaceae family, are distributed across tropical and subtropical regions of the world. *Syzygium oleosum* (F.Muell.) B.Hyland is native to Australia, where it is considered extremely rare, occurring naturally in only a few hectares in northern New South Wales and Queensland [15]. Due to the high content of bioactive compounds in its leafy twigs, this species has gained economic significance to produce EO used in aromatherapy. To support large-scale production and preserve wild populations, commercial plantations have recently been established near Byron Bay (NSW). In these facilities, leafy twigs are hand-harvested, and steam distillation is promptly carried out to ensure optimal EO quality [16].

To the best of current knowledge, ethnomedicinal information on this species is extremely limited. The only well-documented use concerns its fruits, which are consumed raw, prepared as jellies or flavorings, and occasionally employed as a general tonic to promote overall health or as a medicinal pulp for the treatment of earaches [27].

The present study is the first to report the micromorphological and anatomical features of both twigs and leaves, as well as the chemical composition and biological properties of the EO derived from *S. oleosum*. Anatomical analysis revealed that the twigs possess a multilayered cortex characterized by conspicuous secretory cavities arranged in a circular pattern and an abundance of calcium oxalate druses distributed throughout the cortex, in proximity to the phloem, and within the parenchymatous pith. Similar anatomical traits have been documented in other species of the genus, including *Syzygium aromaticum* (L.) Merr. & L.M. Perry [28] and *Syzygium travancoricum* Gamble [29]. The leaf anatomy of *S. oleosum* showed typical features of the Myrtaceae family, such as a dorsiventral mesophyll with numerous secretory cavities and calcium oxalate crystals. Interestingly, *S. oleosum* displayed larger secretory cavities compared to related species, particularly on the adaxial surface, a trend previously reported for other *Syzygium* taxa [30,31,32,33]. Calcium oxalate crystals were predominantly observed as druses, consistent with previous findings in most *Syzygium* species [29,32,33], although other taxa may exhibit prismatic or rhomboidal forms [30,33]. The widespread presence of calcium oxalate crystals in plants is likely associated with several functions, including calcium regulation/storage and protection against herbivory [34]. 

No previous reports on the chemical composition of *S. oleosum* EO are available; thus, comparisons were made with related *Syzygium* species. Literature data confirm that many *Syzygium* EOs contain significant amounts of monoterpene hydrocarbons, with α-pinene, β-pinene, γ-terpinene, limonene, and β-ocimene frequently reported among the most abundant constituents [7,9,35], although in several species, sesquiterpenes such as caryophyllene derivatives and germacrene D represent the dominant fraction [7]. In *S. oleosum* EO, β-pinene and α-pinene were present in high amounts (26.79% and 10.69%, respectively), in line with other species, whereas terpinolene was the most abundant compound (30.79%), exceeding levels typically reported (<15%). Oxygenated monoterpenes were marginal (α-terpineol 2.11%), while eugenol, a major constituent of *S. aromaticum*, was absent [36,37]. Among sesquiterpenes, only β-caryophyllene was detected (0.57%), in contrast to other *Syzygium* EOs where α-/β-caryophyllene, humulene, and germacrene D are common.

The antioxidant and anti-inflammatory effects observed for *S. oleosum* EO and its major monoterpene constituents are consistent with literature data. Numerous studies have highlighted the bioactive potential of α-pinene, β-pinene, γ-terpinene, and terpinolene, often associated with radical-scavenging, metal-chelating, and anti-inflammatory properties. α-Pinene has demonstrated strong antioxidant efficacy, especially in ferric-reducing assays and metal chelation models [38,39]. β-Pinene and terpinolene have been reported to exert moderate to strong radical-scavenging effects in ABTS and FRAP systems; however, under the experimental conditions adopted in the present study, neither compound reached 50% inhibition in TEAC or FRAP assays. This suggests that their antioxidant potential may depend on concentration range, assay sensitivity, and the presence of other constituents in the phytocomplex. γ-Terpinene, with its conjugated double bonds, is noted for potent inhibition of lipid peroxidation [40,41]. Regarding anti-inflammatory activity, α-pinene and γ-terpinene can modulate key pathways, including protein denaturation and protease inhibition [39]. The overall profile of *S. oleosum* EO is therefore consistent with other monoterpene-rich EOs, where compositional complexity contributes to additive or synergistic effects.

The cholinesterase and GABA-T inhibitory activities of *S. oleosum* EO were also evaluated for the first time. Compared with literature data on other *Syzygium* species, the EO exhibited relatively low inhibitory activity. Reported IC_50_ values for acetylcholinesterase inhibition in related taxa ranged from 32.9 to 88.14 µg/mL [17,21] or from 1.5 to 18.2 µL/L [19], sometimes expressed as galantamine equivalents [23]. By contrast, *S. oleosum* EO displayed significantly weaker inhibition [20]. Although α-pinene, known for moderate anticholinesterase potential, was present in considerable amounts, no direct correlation could be established between composition and activity. Indeed, even EOs with different profiles can exert comparable effects [17,18,19,20,21,22,23], suggesting that other factors (e.g., degradation during storage) may contribute [36,37,38]. Additional studies on freshly distilled EO samples from different provenances are needed.

To our knowledge, this is the first investigation of GABA-T inhibition by a *Syzygium* EO. Neither the EO nor its major constituents showed inhibitory activity under the tested conditions. Previous studies have reported modulatory effects of *S. aromaticum* EO—primarily mediated via GABAA receptors rather than GABA-T [42,43]. The absence of inhibition in *S. oleosum* may be linked to its hydrocarbon-rich composition, lacking the oxygenated monoterpenes and phenylpropanoids commonly associated with GABA-T inhibition [44,45,46].

The neurofunctional effects of *S. oleosum* EO were assessed using MEAs. The EO induced concentration-dependent inhibition of neuronal network activity without cytotoxicity, with %Spike_B being the most sensitive parameter. This pattern parallels observations from *Citrus lumia* EO, though the latter exhibited lower IC_50_ values due to its high D-limonene content [47]. The compositional differences likely explain the relatively weaker potency of *S. oleosum*. Nonetheless, the selective effect on burst structure resembles that of other monoterpene-rich EOs, including *Lavandula angustifolia* and *Coriandrum sativum* [48]. Overall, the data suggest a neuromodulatory rather than neurotoxic profile.

The overall biological profile of *S. oleosum* EO, characterized by moderate antioxidant and anti-inflammatory activities together with non-cytotoxic neuromodulatory effects, suggests potential applications in areas where mild but broad-spectrum bioactivity is desirable. In particular, its composition, rich in monoterpene hydrocarbons, could support use in aromatherapy, functional foods, or cosmetic formulations aimed at counteracting oxidative stress and low-grade inflammation. Moreover, the observed neuromodulatory properties, coupled with the absence of cytotoxicity, point to possible nutraceutical or pharmaceutical interest as a safe neuromodulatory agent, warranting further preclinical investigation.

## 4. Materials and Methods

### 4.1. Plant Material and Essential Oil

**Figure 6:**
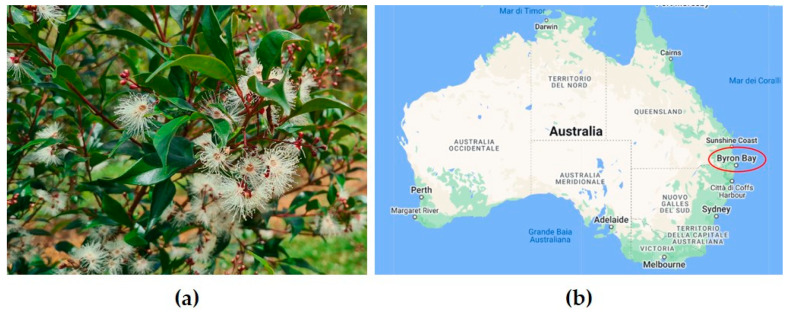
(**a**) *S. oleosum* growing in its environment. (**b**) Map of Australia showing where *S. oleosum* is cultivated (red circle).

Small branches bearing juvenile foliage of *Syzygium oleosum* (F.Muell.) B.Hyland were collected in May 2023 from commercial plantations near Byron Bay (NSW, Australia) (Figure 6). The plantation consists of trees cultivated in rows and maintained at a maximum height of approximately 4 m. The sampled trees were between 15 and 20 years old. The EO was obtained by steam distillation (2 h) of freshly harvested leaves and young twigs. The plant material was hand-harvested and distilled immediately after collection to preserve EO quality. No wild trees were harvested for this study.

### 4.2. Micromorphological Analyses

Micromorphological analyses were carried on leaves hand-made cross sections, obtained by using a double-edged razor blade, and on small specimens of leaf surface. Both samples were cleared with an aqueous solution of chloral hydrate and mounted in a chloral hydrate–glycerol solution to prevent crystallization of the reagent during observations, according to Jackson and Snowdon [49]. The sections were observed under transmission light microscopy to carry out a morphometric analysis of the secretory cavities. For this purpose, the polar and equatorial axes of the cavities were measured by using the Windows Microscope ToupView Package 2025-07-29 (ToupTek Photonics, Hangzhou, China). Afterwards, to detect the presence of lipophilic substances inside secretory tissues some cleared sections were treated with Sudan III (Merck, Darmstadt, Germany) and other fresh sections were stained with Fluorol Yellow 088 [50]. Observations were made with a Leica DM 2000 fluorescence microscope equipped with an H3 filter (excitation filter BP 420–490 nm) (Leica Microsystems, Wetzlar, Germany) and with a ToupCam Digital Camera, CMOS Sensor 3.1 MP resolution (ToupTek Photonics, Hangzhou, China). Furthermore, polarized light was used to detect the presence and distribution of crystals within the plant tissues.

For a more detailed analysis of the epidermal surface and of the oil gland cavities within the mesophyll, leaves were also observed using SEM. For this purpose, leaves were fixed in 70% ethanol–FineFix working solution (Milestone s.r.l., Bergamo, Italy) for 24 h at 4 °C, dehydrated through a series of increasing ethanol solutions (70, 80, 90 and 100%) for 1 h each [51], and critical-point-dried in CO_2_ (CPD, K850 2M Strumen-ti s.r.l., Rome, Italy). Finally, sections and epidermal surfaces of the dried leaf samples were mounted on aluminum stubs using glued carbon tabs, sputter-coated with 10 nm gold [52], and observed with a Vega3 Tescan LMU SEM (Tescan USA Inc., Cranberry Twp, PA, USA) equipped with the Energy Dispersive X-ray Spectroscopy (EDS) (Apollo, Tescan USA Inc., Cranberry Twp, PA, USA), operating at an accelerating voltage of 20 kV. EDS was used to identify the elemental composition of crystals [53].

### 4.3. Gas Chromatography with Flame Ionization Detection (GC-FID) and Gas Chromatography–Mass Spectrometry (GC-MS) Analysis

GC-FID analysis was performed using a Perkin-Elmer Sigma 115 gas chromatograph (Waltham, MA, USA) and a non-polar HP-5 MS fused silica capillary column (30 m × 0.25 mm i.d.; 0.25 μm film thickness). The injector and detector temperatures were set at 250 °C and 290 °C, respectively. The oven temperature program was as follows: isothermal at 40 °C for 5 min, then increased at a rate of 2 °C/min up to 270 °C, followed by an isothermal hold at 270 °C for 20 min. Analyses were also conducted on a polar HP Innowax column (50 m × 0.20 mm i.d.; 0.25 μm film thickness) under the same conditions to support compound identification. In both cases, helium was used as the carrier gas at a constant flow rate of 1.0 mL/min.

GC-MS analyses were carried out using an Agilent 6850 Series II system (Santa Clara, CA, USA), equipped with a DB-5 fused silica capillary column (30 m × 0.25 mm i.d.; 0.25 μm film thickness) and coupled to an Agilent 5973 Mass Selective Detector (Santa Clara, CA, USA). The mass spectrometer was operated with an electron impact ionization energy of 70 eV and an ion multiplier voltage of 2000 V. Mass spectra were acquired over a mass range of 40–500 amu at a rate of five scans per second. The chromatographic conditions were identical to those used in the GC-FID analysis, with the transfer line temperature set at 295 °C.

Compound identification was achieved by comparing the experimentally determined Kovats retention indices (KI), calculated relative to a homologous series of *n*-alkanes (C10–C35) under the same conditions, with those reported in the literature [54,55,56,57]. Additionally, the mass spectral data were interpreted through comparison with those of authentic standards and with reference spectra available in the NIST 14 and Wiley 257 mass spectral libraries [58]. For selected compounds, identification was further confirmed by co-injection with pure reference substances. The relative concentrations of the components were calculated using peak area normalization, without applying correction factors.

### 4.4. In Vitro Cell-Free Assays

#### 4.4.1. Antioxidant and Anti-Inflammatory Assays

The antioxidant and anti-inflammatory properties of *S. oleosum* and its major bioactive compounds were evaluated using different in vitro assays based on different mechanisms and reaction environments according to Smeriglio et al. [59]. Results were expressed as the concentration required to inhibit 50% of the oxidative or inflammatory activity (IC_50_, µg/mL), with corresponding 95% confidence limits (C.L.) calculated using the Litchfield and Wilcoxon method with PHARM/PCS software (version 4; Consulting, Wynnewood, PA, USA).

##### TEAC Assay

To generate the 2,2′-azino-bis (3-ethylbenzothiazoline-6-sulfonic acid) (ABTS) radical cation solution, ABTS^+^ (1.7 mM) was incubated with potassium persulfate (4.3 mM) in a 1:5 (*v*/*v*) ratio in the dark at room temperature (RT) for 12 h. The resulting solution was diluted to achieve an absorbance of 0.7 ± 0.02 at 734 nm. For the assay, 10 µL of either EO (ranging from 0.313 to 5 mg/mL), its main bioactive compounds (0.096–1.54 mg/mL, 0.084–1.34 mg/mL, 0.033–0.535 mg/mL, and 0.031–0.493 mg/mL for terpinolene, β-pinene, α-pinene, and γ-terpinene) or Trolox (0.625–10 µg/mL) was added to 200 µL of the ABTS solution and incubated at RT for 6 min. The absorbance at 734 nm was measured using a Multiskan™ GO UV-Vis microplate reader (Thermo Scientific, Waltham, MA, USA).

##### FRAP Assay

In this assay, 10 µL of EO (ranging from 0.313 to 5 mg/mL), its main bioactive compounds (0.096–1.54 mg/mL, 0.084–1.34 mg/mL, 0.033–0.535 mg/mL, and 0.031–0.493 mg/mL for terpinolene, β-pinene, α-pinene, and γ-terpinene) or Trolox (1.25–10 µg/mL) was mixed with 200 µL of FRAP reagent, pre-incubated at 37 °C. The reagent consisted of 10 mM 2,4,6-Tris (2-pyridyl)-s-triazine (TPTZ) in 40 mM HCl, 20 mM FeCl_3_·6H_2_O, and 300 mM acetate buffer (pH 3.6). The mixture was incubated for 4 min at RT in the dark before measuring absorbance at 593 nm with the same microplate reader used in Section TEAC Assay.

##### ORAC Assay

ORAC activity was evaluated by combining 20 µL EO (ranging from 0.003 to 0.05 mg/mL), its main bioactive compounds (0.0010–0.0154 mg/mL, 0.0008–0.0134 mg/mL, 0.0003–0.0053 mg/mL, and 0.003–0.0049 mg/mL for terpinolene, β-pinene, α-pinene, and γ-terpinene) or Trolox (0.25–2 µg/mL) with 120 µL of fluorescein solution (117 nM). After a 15-min pre-incubation at 37 °C, 60 µL of freshly prepared 2,2′-azobis (2-amidinopropane) dihydrochloride (AAPH) solution (40 mM) was added to initiate peroxyl radical formation. Fluorescence decay was monitored every 30 s for 90 min using excitation at 485 nm and emission at 520 nm on a FLUOstar Omega microplate reader (BMG LABTECH, Ortenberg, Germany).

##### BCB Assay

In brief, 200 µL of EO (0.094–1.5 mg/mL), its main bioactive compounds (0.0289–0.4619 mg/mL, 0.0251–0.4019 mg/mL, 0.010–0.1604 mg/mL, and 0.0092–0.1479 mg/mL for terpinolene, β-pinene, α-pinene, and γ-terpinene), butylated hydroxytoluene (BHT, 1 mg/mL), or blank (0.1% DMSO) was added to 5 mL of β-carotene emulsion, prepared from 250 µL of β-carotene in ethyl acetate (1 mg/mL), 4 µL linoleic acid, and 40 µL Tween-40. An emulsion lacking β-carotene served as negative control. The mixtures were incubated at 50 °C for 2 h, and absorbance at 470 nm was measured at 20 min intervals using the same instrument as in Section TEAC Assay.

##### ICA Assay

In brief, 25 µL of FeCl_2_·4H_2_O (2 mM) was mixed with 50 µL EO (0.010–0.160 µg/mL), its main bioactive compounds (0.0031–0.0493 mg/mL, 0.0027–0.0429 mg/mL, 0.0011–0.0171 mg/mL, and 0.0009–0.0158 mg/mL for terpinolene, β-pinene, α-pinene, and γ-terpinene), or EDTA (1.75–14 µg/mL). After 5 min at RT, 50 µL of ferrozine solution (5 mM) and deionized water were added to bring the total volume to 1.5 mL. Following vortexing and a 10 min incubation, absorbance at 562 nm was recorded using the plate reader described in Section TEAC Assay.

##### ADA Assay

EO (0.25–4 mg/mL) and its main bioactive compounds (0.0769–1.2316 mg/mL, 0.0669–1.0716 mg/mL, 0.0267–0.4276 mg/mL, and 0.0247–0.3944 mg/mL for terpinolene, β-pinene, α-pinene, and γ-terpinene) were combined with 0.4% BSA and PBS buffer (pH 5.3) at a 4:5:1 ratio. Diclofenac sodium (3.0–24.0 µg/mL) was included as a reference standard. Absorbance at 595 nm was measured before and after 30 min of incubation at 70 °C in a shaking water bath, using the UV–Vis reader mentioned in Section TEAC Assay.

##### PIA Assay

For the assay, 200 µL of EO (0.15–2.4 mg/mL), its main bioactive compounds (0.04619–0.7389 mg/mL, 0.0402–0.6429 mg/mL, 0.0160–0.2566 mg/mL, and 0.0148–0.2366 mg/mL for terpinolene, β-pinene, α-pinene, and γ-terpinene) or diclofenac sodium (5–80 µg/mL) was combined with 12 µL trypsin (10 µg/mL), 188 µL Tris-HCl buffer (20 mM, pH 7.5), and 200 µL of casein (0.8%). After a 20 min incubation at 37 °C, the reaction was halted with 400 µL of 2 M perchloric acid, followed by centrifugation at 3500 × *g* for 10 min. The absorbance of the supernatant was measured at 280 nm using a UV-1601 spectrophotometer (Shimadzu, Kyoto, Japan).

### 4.5. Cholinesterases Inhibition

The cholinesterase inhibitory activity was evaluated according to the method described by Zheng and co-workers [60].

The assay was carried out in a total volume of 1 mL, containing 415 μL of 0.1 M Tris–HCl buffer (pH 8.0), 10 μL of the EO buffer solution or of the major compounds (dissolved in 0.1% DMSO) at various concentrations (ranging from 25 to 1 mg/mL for the acetylcholinesterase [AChE] assay, and from 20 to 1 mg/mL for the butyrylcholinesterase [BChE] assay), and 25 μL of enzyme solution containing 0.28 U/mL of AChE or BChE. The mixture was incubated for 15 min at 37 °C.

Subsequently, 75 μL of a 1.83 mM solution of acetylthiocholine iodide (AChI) or butyrylthiocholine iodide (BChI) and 475 μL of DTNB (5,5′-dithiobis-(2-nitrobenzoic acid)) were added, and the reaction mixture was further incubated for 30 min at 37 °C. Absorbance was measured at 405 nm using a spectrophotometer (Thermo Fisher Scientific, Vantaa, Finland). Galantamine was used as a positive control.

### 4.6. GABA-Transaminase (GABA-T) Inhibition

The GABA-T inhibition assay was carried out according to Choi et al. [61] with some modifications.

Fresh reagents were prepared in Milli-Q water to obtain the following stock concentrations: GABA (100 μg/mL), α-ketoglutarate (95 μg/mL) and NADP^+^ (16 mM). Stock solutions of *S. oleosum* EO (1.25–20 mg/mL) and its major bioactive compounds based on the essential oil composition (0.385–6.158 mg/mL, 0.335–5.358 mg/mL, 0.134–2.138 mg/mL, and 0.123–1.972 mg/mL for terpinolene, β-pinene, α-pinene, and γ-terpinene, respectively) were prepared in 0.1% DMSO.

The reaction buffer consisted of 0.1 M potassium pyrophosphate (pH 8.6), while GABA transaminase (GABAse) from *Pseudomonas fluorescens* (10 U/mL) was solubilized in 75 mM phosphate buffer (pH 7.2) containing 25% (*v*/*v*) glycerol, according to the manufacturer’s instructions.

Each test solution (final volume: 800 μL) was composed of 100 μL of GABA, α-ketoglutarate, and NADP^+^ solutions, 10 μL of 2-mercaptoethanol, 20 μL of 0.1% DMSO (control), *S. oleosum* EO or pure bioactive compounds, and potassium pyrophosphate buffer to volume. Reactions were initiated by the addition of 20 μL of GABAse (10 U/mL). The enzymatic assay consisted of two coupled steps: first, GABA was transaminated to succinic semialdehyde by GABA-T; this intermediate was then oxidized to succinate by succinic semialdehyde dehydrogenase (SSDH), with stoichiometric reduction of NADP^+^ to NADPH. The formation of NADPH was monitored spectrophotometrically at 340 nm for 30 min at room temperature (25 °C). Results were expressed as NADPH formation kinetics and, by selecting the linear portion of the curve (first-order phase, 10 min), as residual GABA-T activity (%) in the presence of each inhibitor. The absorbance value of the control was set to 100% activity, and activity of each concentration of *S. oleosum* EO or pure bioactive compounds was measured relative to the control. Taurine was used as a positive GABA-T inhibitor control according to Salaiman et al. [62].

### 4.7. In Vitro Cell-Based Assays

#### 4.7.1. Primary Neuronal Cultures

Primary neuronal cultures were prepared from the cerebral cortices of E17 Wistar SPF rat embryos (both sexes), following established protocols [63]. Cortical tissues were mechanically dissociated in 5 mL of calcium- and magnesium-free Hank’s Balanced Salt Solution (HBSS, Thermo Fisher Scientific, Waltham, MA, USA) using two fire-polished Pasteur pipettes of decreasing diameter. After sedimentation, the supernatant was discarded, and the cell pellet was gently resuspended in Neurobasal medium (Thermo Fisher Scientific) supplemented with 2% B27 (Thermo Fisher Scientific) and 1% glutamine (Sigma-Aldrich, Milan, Italy). The suspension was then appropriately diluted and plated onto 96-well plates for viability assays or on MEA chips for electrophysiological recordings.

#### 4.7.2. Cell Viability Assay

Neurons were seeded at a density of 3 × 10^3^ cells per well into 96-well plates pre-coated with 0.1% polyethyleneimine (PEI) and maintained in NB medium supplemented with 2% B27 and 1% Glutamax-I. Cultures were kept at 37 °C in a humidified 5% CO_2_ incubator, with half the medium replaced three times weekly. After 21 days in vitro (DIV), cultures were exposed to increasing concentrations of EO (5–200 μg/mL) for 2 h. Following exposure, 20 µL of MTT solution (3-(4,5-dimethylthiazol-2-yl)-2,5-diphenyltetrazolium bromide; Sigma-Aldrich) were added to each well. After an additional 2 h incubation at 37 °C, the formazan crystals were solubilized with DMSO, and absorbance was read at 570 nm. Cell viability was expressed as a percentage relative to untreated controls.

#### 4.7.3. Electrophysiological Recordings and Data Analysis

For electrophysiological experiments, 50,000–60,000 neurons were seeded at the center of PEI-coated 60-electrode MEA chips (60MEA200/30iR-Ti-gr; Multi Channel Systems, MCS GmbH, Reutlingen, Germany) and allowed to adhere for 1 h. Subsequently, 1 mL of pre-warmed NB medium supplemented with 2% B27 and 1% Glutamax-I was added to each chip. Cultures were maintained for 4 to 6 weeks in a humidified incubator (37 °C, 5% CO_2_), with partial medium replacement three times per week.

Recordings were performed using the MEA120 INV system (MCS, Reutlingen, Germany), with chips connected to the amplifier (Gain: 1000×) and data acquired at a 10 kHz sampling rate using MC_Rack software (v. 4.4.1.0). Signals were band-pass filtered (60–4000 Hz) to eliminate background noise, and only spikes exceeding 5.5× the standard deviation of the baseline noise were considered. A temperature controller (TC02, MCS GmbH) maintained the cultures at 37 °C during recordings. EO was applied cumulatively in the range of 5–200 μg/mL.

Analysis of neuronal activity was performed using NeuroExplorer software (v. 4.135; Nex Technologies, CO, USA). The following parameters were extracted: MFR: Mean firing rate (spikes/s); MBR: Mean burst rate (bursts/min); %Spike_B: Percentage of spikes within bursts; MBD: Mean burst duration (s); MISI_B: Mean inter-spike interval within bursts (s).

Burst detection was defined using the following parameters: bin size = 1 s; max start interval = 0.01 s; max end interval = 0.075 s; minimum inter-burst interval = 0.1 s; minimum burst duration = 0.02 s; minimum spikes per burst = 4. Only channels exhibiting >2 bursts per minute were included in the analysis.

IC_50_ values were calculated by fitting normalized concentration–response data with a four-parameter logistic function using SigmaPlot 8 (Jandel Scientific, Erkrath, Germany):

*f*(*x*) = Max + Min − Max1 + (*ε*/*x*)*β*

where *x* is the EO concentration, Min and Max represent the asymptotic extremes of the curve, *ε* is the inflection point corresponding to the IC_50_, and *β* denotes the slope at that point.

### 4.8. Statistical Analysis

Results are presented as mean ± standard deviation (S.D.) or standard error of the mean (S.E.M.) as appropriate. For in vitro cell-free assays, data were obtained from three independent experiments, each performed in triplicate. For cell-based assays, data were derived from eight and twelve independent experiments, also conducted in triplicate.

Statistical analyses were performed using one-way ANOVA followed by Tukey’s post hoc test for phytochemical and cell-free assays, and the Holm–Sidak method for cell-based assays. All analyses were conducted using SigmaPlot version 12.0. Differences were considered statistically significant at *p* < 0.05.

## 5. Conclusions

The present study provides the first detailed characterization of the leafy twigs of *Syzygium oleosum* (F.Muell.) B.Hyland, highlighting unique micromorphological traits and the monoterpene-rich composition of their EO. This EO exhibited moderate antioxidant and anti-inflammatory activities, together with weak cholinesterase inhibition and no effect on GABA-T. Electrophysiological assays further demonstrated a concentration-dependent neuromodulatory effect without evidence of cytotoxicity, pointing to a safe pharmacological profile. Importantly, the scarcity of ethnomedicinal information on this species underscores the novelty of these findings and their relevance for expanding knowledge within the genus *Syzygium*. Taken together, the results indicate that *S. oleosum* EO could represent a promising natural resource for health-related applications, particularly as a mild anti-inflammatory or neuromodulatory agent. Future studies should explore its mechanisms of action, evaluate its efficacy in in vivo models, and assess its potential incorporation into nutraceutical, cosmetic, or aromatherapy formulations.

## Figures and Tables

**Table 1:** Dimensions of secretory cavities of *Syzygium oleosum* (F.Muell.) B.Hyland, measured from LM micrographs, expressed as mean ± standard deviation (*n* = 30).

Epidermis	Polar Diameter (μm)	Equatorial Diameter (μm)
Upper	119.2 ^a^ ± 22.3	133.4 ^a^ ± 24.5
Lower	88.7 ^b^ ± 26.0	102.5 ^b^ ± 32.0

^a,b^ Means followed by different letters within columns are significantly different at *p* < 0.05 according to a one-way ANOVA.

**Table 2:** Chemical Composition of the EO of *Syzygium oleosum* (F.Muell.) B.Hyland.

N.	Compound	%	Ki ^a^	Ki ^b^	Identification ^c^
1	α-Thujene	4.07	859	1027	1,2
2	α-Pinene	10.69	864	1025	1,2,3
3	β-Pinene	26.79	904	1110	1,2,3
4	β-Myrcene	3.79	920	1161	1,2
5	α-Phellandrene	1.53	927	1168	1,2,3
6	α-Terpinene	1.71	939	1178	1,2,3
7	*p*-Cymene	1.35	947	1270	1,2
8	Limonene	3.35	950	1198	1,2,3
9	β-Ocimene	0.32	972	1250	1,2
10	γ-Terpinene	9.86	981	1245	1,2,3
11	Terpinolene	30.79	1009	1282	1,2,3
12	Linalool	0.33	1018	1543	1,2,3
13	Bicyclo[2.2.1]hept-2-en-7-ol	0.48	1059	1709	1,2
14	Terpinen-4-ol	2.26	1089	1601	1,2,3
15	α-Terpineol	2.11	1096	1694	1,2
16	β-Caryophyllene	0.57	1303	1588	1,2
	**Total**	100.00			
Monoterpene hydrocarbons		94.25			
Oxygenated monoterpenes Sesquiterpene hydrocarbons		5.18			
		0.57			

^a,b^ The Kovats retention indices are relative to a series of *n*-alkanes (C10–C35) on the apolar DB-5 and the polar HP Innowax capillary columns, respectively. ^c^ Identification method: 1 = comparison of the Kovats retention indices with published data, 2 = comparison of mass spectra with those listed in the NIST 02 and Wiley 275 libraries and with published data, and 3 = co-injection with authentic compounds.

**Table 3:** Antioxidant and anti-inflammatory activity of EO of *Syzygium oleosum* (F.Muell.) B.Hyland, in comparison with its most abundant bioactive compounds (α-pinene, β-pinene, γ-terpinene, and terpinolene) and reference standards. Pure bioactive compounds were tested at the concentration naturally present within the EO with reference to its concentration range tested. Results, which represent the mean of three independent experiments in triplicate (*n* = 3), are expressed as the concentration inhibiting 50% of the activity (IC_50_) with 95% confidence limits (between brackets).

Assay	EO	α-Pinene	β-Pinene	γ-Terpinene	Terpinolene	RS ^a^
	mg/mL					µg/mL
TEAC	4.95 (4.02–6.09)	n.r. ^b^	n.r.	n.r.	n.r.	3.78 (1.48–9.67) ***
FRAP	3.15 (1.99–5.00)	n.r.	n.r.	n.r.	n.r.	3.72 (1.60–8.66) ***
ORAC	0.02 (0.02–0.03)	n.r.	n.r.	n.r.	0.01 (0.00–0.04)	0.68 (0.22–82.17) ***
BCB	0.24 (0.19–0.31)	n.r.	n.r.	0.02 (0.02–0.03)	0.21 (0.10–0.45)	0.35 (0.17–80.58) ***
ICA	0.12 (0.10–0.14)	0.5 (0.30–0.83)	0.03 (0.01–0.13)	n.r.	n.r.	5.72 (2.32–87.13) ***
ADA	3.53 (2.65–4.72)	n.r.	0.36 (0.29–0.45)	0.11 (0.09–0.14)	0.32 (0.26–0.39)	17.05 (13.94–20.85) ***
PIA	5.93 (2.72–12.92)	0.13 (0.11–0.15)	n.r.	n.r.	1.87 (1.53–2.29)	28.50 (13.31–861.04) ***

^a^ RS, Reference standard; trolox for FRAP, TEAC, and ORAC assay; BHT for BCB; EDTA for ICA; diclofenac sodium for ADA and PIA; *** *p* < 0.001 vs. EO and all bioactive compounds; ^b^ n.r., IC_50_ not reached.

**Table 4:** Anticholinesterase activity of EO of *Syzygium oleosum* (F.Muell.) B.Hyland, in comparison with its most abundant bioactive compounds (α-pinene, β-pinene, γ-terpinene, and terpinolene) and the reference standard (galantamine). Pure bioactive compounds were tested at the concentration naturally present in the EO, with reference to its concentration range tested. The results are expressed as the concentration that inhibits 50% of the enzimatic activity (IC_50_) ± standard deviation.

	AChE (mg/mL)	BChE (mg/mL)
EO	19.4 ± 0.6 ^a^	15.9 ± 0.1 ^a^
α-Pinene	0.05 ± 0.01 ^d^	n.r.
β-Pinene	n.r.	n.r.
γ-Terpinene	n.r.	n.r.
Terpinolene	1.0 ± 0.05 ^c^	1.4 ± 0.05 ^c^
Galantamine	(2.3 ± 0.2) ^b^ × 10^−3^	(1.4 ± 0.07) ^b^ × 10^−2^

AChE, acetylcholinesterase; BChE, butyrilcolinesterase. Mean ± SD = indicates the mean value of the three experiments and the value of the standard deviation. Different letters (a–d) next to means in the same column indicate that those values belong to statistically different groups (*p* < 0.05), according to a two-way ANOVA followed by Tukey’s post hoc test. The letters have no intrinsic meaning but serve only to identify groups between which there are significant differences. Galantamine was used as reference compound. n.r.: IC_50_ not reached.

## Data Availability

The original contributions presented in this study are included in the article. Further inquiries can be directed to the corresponding author.

## Supplementary Materials

The following supporting information can be downloaded at https://www.mdpi.com/article/10.3390/plants14162605/s1. Figure S1. GC chromatogram of the essential oil of *Syzygium oleosum* (F.Muell.) B.Hyland, displaying the separation profile of its volatile constituents.

## Abbreviations

The following abbreviations are used in this manuscript:

ABTS	2,2′-Azino-bis(3-ethylbenzothiazoline-6-sulfonic acid)
ADA	Anti-denaturation assay
ANOVA	Analysis of Variance
BCB	β-Carotene Bleaching
BChE	Butyrylcholinesterase
CPD	Critical Point Drying
DTNB	5,5′-Dithiobis-(2-nitrobenzoic acid)
EDS	Energy Dispersive Spectroscopy
EO	Essential Oil
FRAP	Ferric-Reducing Antioxidant Power
GABA	Gamma-Aminobutyric Acid
GABA-T	GABA-Transaminase
GC	Gas Chromatography
GC-FID	Gas Chromatography with Flame Ionization Detection
GC-MS	Gas Chromatography–Mass Spectrometry
HBSS	Hank’s Balanced Salt Solution
IC50	Half Maximal Inhibitory Concentration
ICA	Iron Chelating Activity
MBD	Mean Burst Duration
MBR	Mean Burst Rate
MEA	Microelectrode Array
MFR	Mean Firing Rate
MISI_B	Mean Inter-Spike Interval within Bursts
MTT	3-(4,5-Dimethylthiazol-2-yl)-2,5-diphenyltetrazolium bromide
ORAC	Oxygen Radical Absorbance Capacity
PBS	Phosphate Buffered Saline
PEI	Polyethyleneimine
PIA	Protease Inhibitory Activity
RT	Room Temperature
SD	Standard Deviation
SEM	Scanning Electron Microscopy
SSDH	Succinic Semialdehyde Dehydrogenase
TEAC	Trolox Equivalent Antioxidant Capacity
TPTZ	2,4,6-Tripyridyl-s-triazine
