# Supplementary figures and images for: Syzygium oleosum (F.Muell.) B.Hyland (Myrtaceae), an Unexplored Australian Species: Anatomical and Micromorphological Study of Leafy Twigs, and Characterization and Biological Activity of Their Essential Oil
Source: Plants (Basel). 2025 Aug 21;14(16):2605. doi: 10.3390/plants14162605 (PMC12389591; doi:10.3390/plants14162605)

Chromatogram Plot Report

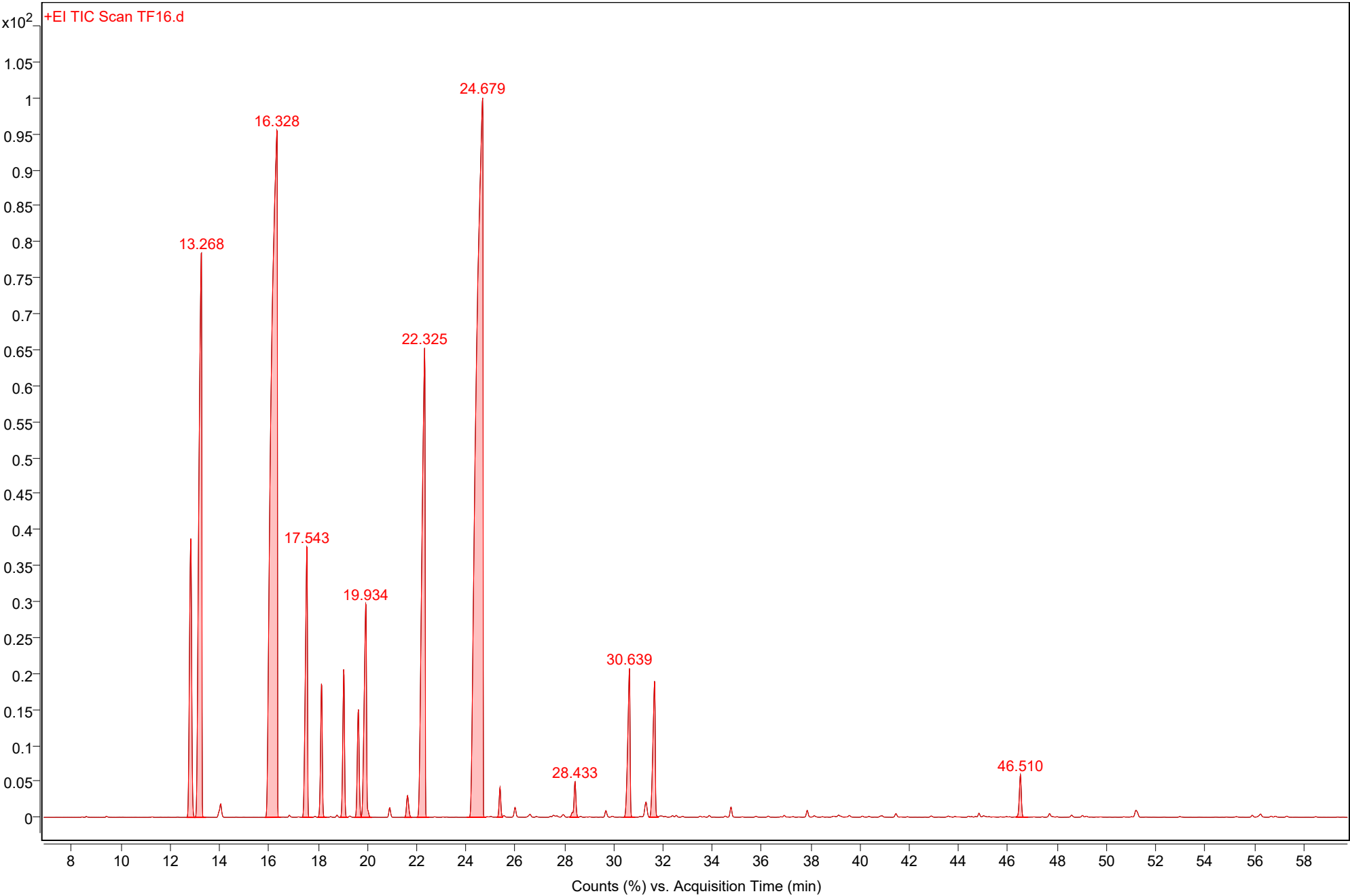

Supplement: Supplementary file 1 [file plants-14-02605-s001.zip › plants-3810525-supplementary.pdf]
